# Supplementary material for: Guiding the design of well-powered Hi-C experiments to detect differential loops
Source: Bioinform Adv. 2023 Oct 16;3(1):vbad152. doi: 10.1093/bioadv/vbad152 (PMC10645293; doi:10.1093/bioadv/vbad152)
Supplement: vbad152_Supplementary_Data [file vbad152_supplementary_data.zip › HPOW_Supplemental_Figs.docx]

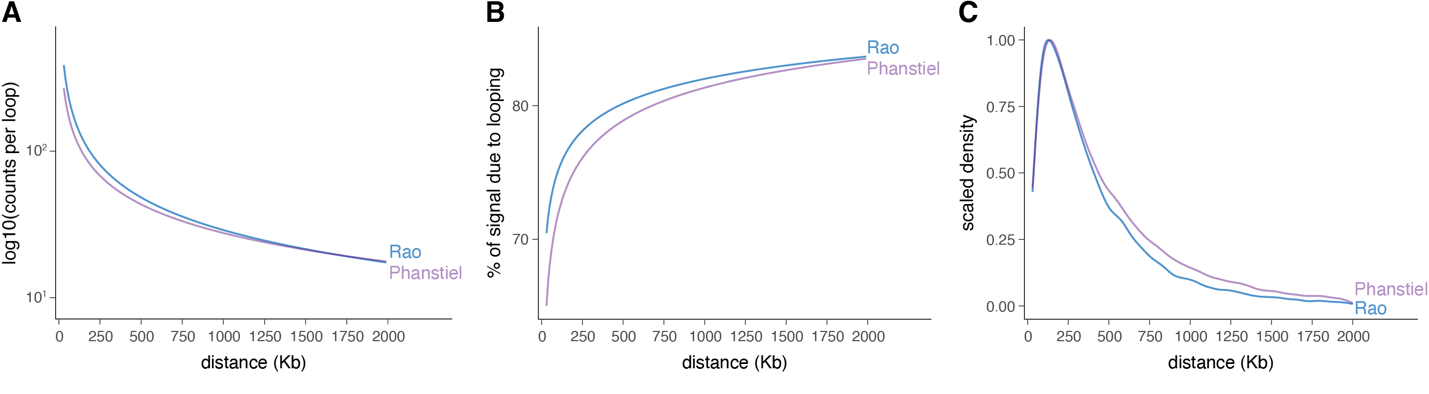


**Figure S1.** **The effect of loop size on counts, loop composition, and quantity of loops from two Hi-C data sets.  (A)** Median counts vs loop size are plotted for loops from THP-1 cells (Phanstiel et al.) and GM12878 cells (Rao et al.). **(A)** Median percent of signal due to looping vs loop size is plotted for loops from THP-1 cells (Phanstiel et al.) and GM12878 cells (Rao et al.). **(C)** Distribution of loop sizes identified from THP-1 cells (Phanstiel et al.) and GM12878 cells (Rao et al.).

**
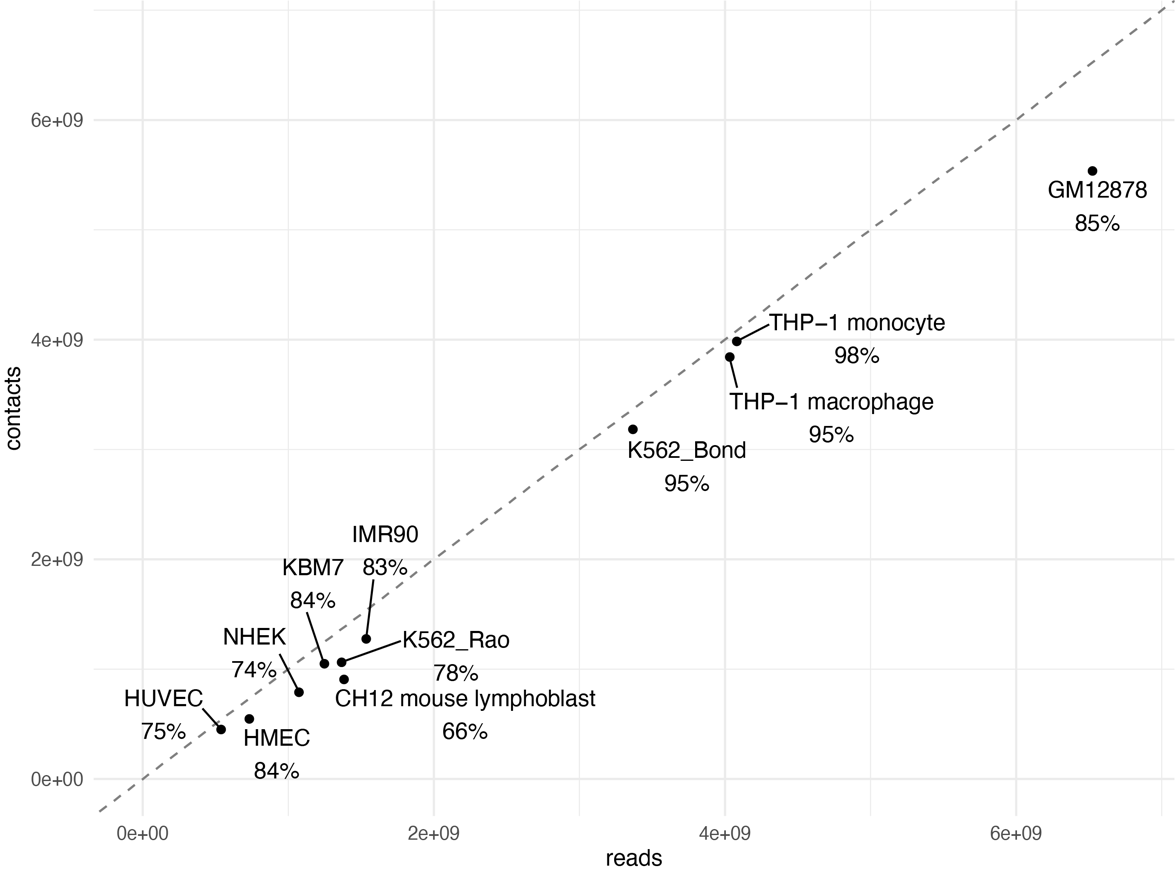
Figure S2.** **Percent of sequenced reads that are unique contacts for various cell lines.** The total reads and contacts and their percentages for the Hi-C datasets in Rao et al., 2014 (CH12 mouse lymphoblast, GM12878, HMEC, HUVEC, IMR90, K562, KBM7, and NHEK), Phanstiel et al., 2017 (THP-1 monocyte and THP-1 macrophage), and Bond et al., 2022 (K562). The GM12878 dataset was chosen for this study as it is the most deeply sequenced.


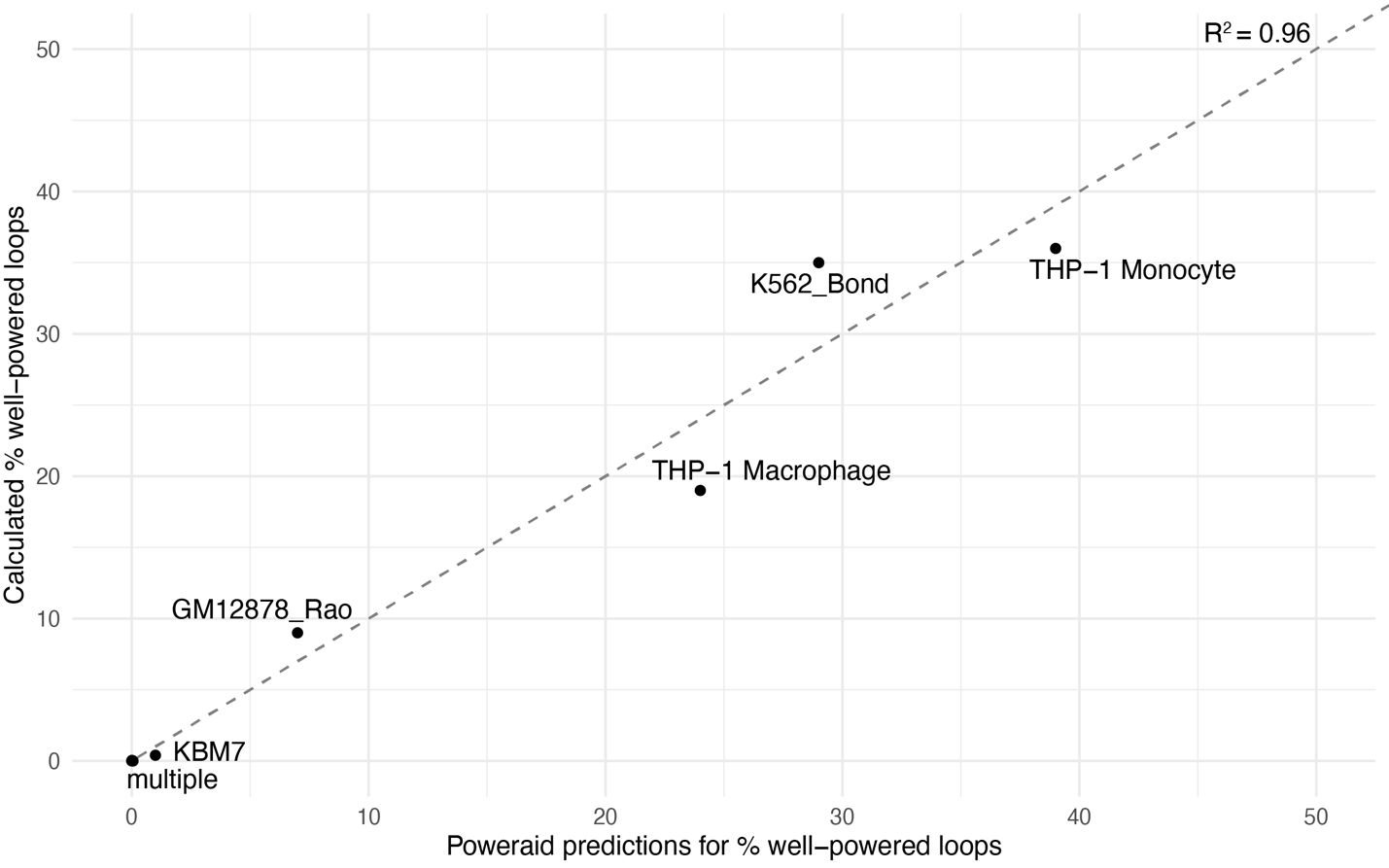


**Fig S3. Predicted percentages of well-powered loops using Hi-C Poweraid compared to the calculated percentage of well-powered loops using Hi-C data from various cell lines.** The percentage of loops with a power greater than or equal to 0.8 to detect a 2-fold change in looping (“well-powered loops”) was calculated using the unnormalized counts for each of the Hi-C datasets with 2 or more replicates from Rao et al., 2014, Phanstiel et al., 2017, and Bond et al., 2022. The percentage of well-powered loops for each of these datasets was also predicted using Hi-C Poweraid with parameters most closely matching those of the respective dataset, using subsampled counts from the GM12878 dataset. The “multiple” datasets represented here all had percentages at or near 0% and include IMR90, HMEC, K562_Rao, KBM7, and CH12 mouse lymphoblast.

**Fig S4. Percentage of well-powered loops for 5Kb and 10Kb resolution. (A)** Figure 3A is recreated here, using 5Kb loops instead of 10Kb loops. To reach 50% or more well-powered loops for an experiment with a dispersion of 0.001, at least 15 billion total contacts are required. This is mostly due to the lower counts per 5Kb bin compared to a 10Kb bin for the same total sequencing depth. **(B)** Figure 3A is duplicated here for ease of comparison, representing the percent of well-powered loops by total sequencing depth for 10Kb loops.


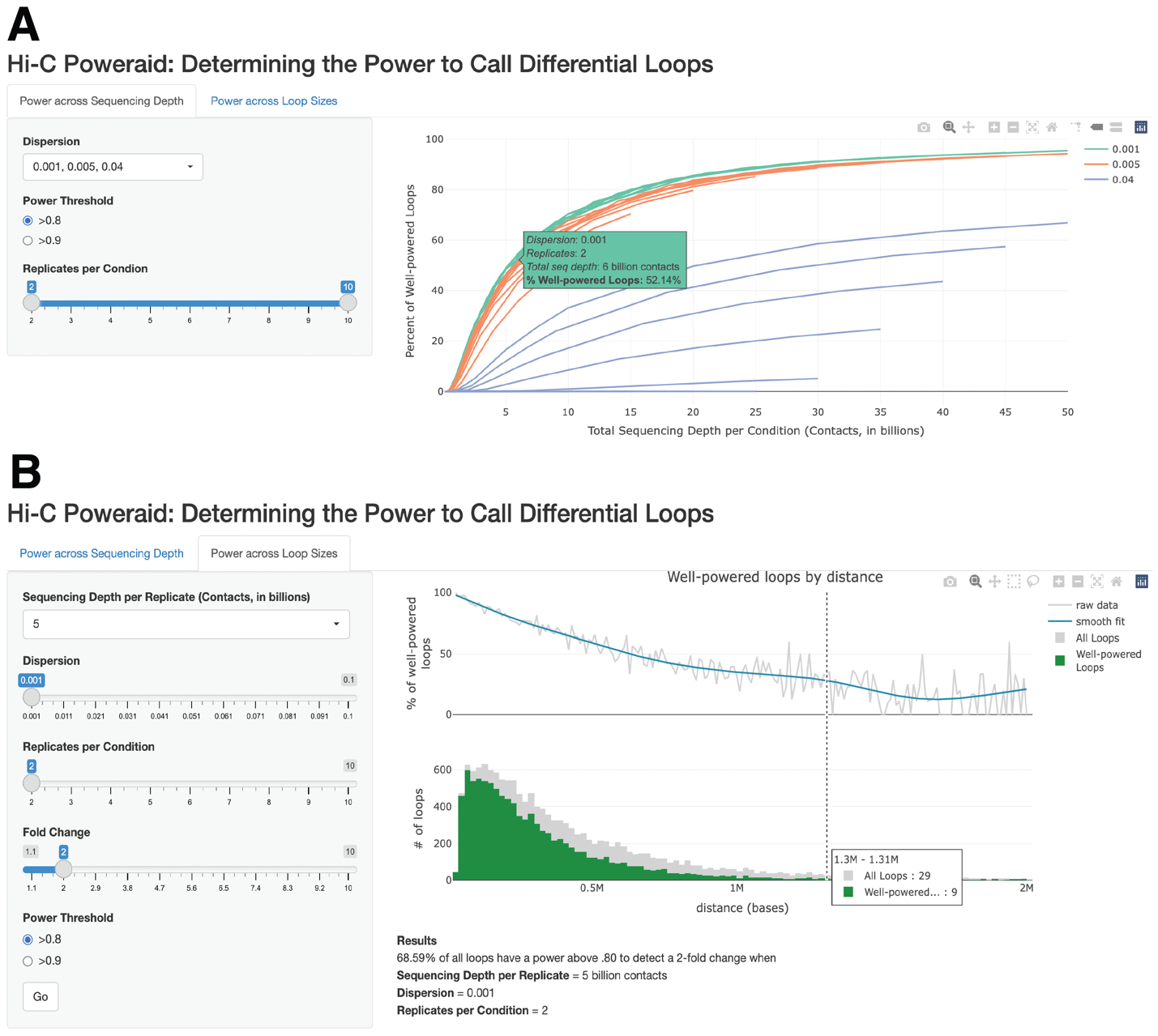


**Fig S5. Screenshots of Hi-C Poweraid.** **(A)** **Power Across Sequencing Depth Tab.** This tab of Hi-C Poweraid allows users to recreate Figure 3A with the parameters of their choice, representing the percent of well-powered loops by total sequencing depth for 10Kb loops. Multiple dispersions can be selected, and the range of replicates can also be adjusted. The hover effect is also displayed for the point with a 0.001 dispersion, 2 replicates, and 6 billion contacts. The icons in the top right of the plot highlight the options built into the plotly framework, such as panning, zooming, and saving the plot. **(B) Power Across Loop Sizes Tab.** This tab of Hi-C Poweraid allows users to investigate further into one set of parameters, representing one Hi-C experiment. One value can be selected for Sequencing Depth per Replicate, Dispersion, Replicates per Condition, Fold Change, and Power Threshold. The resulting plots display the percentage of loops that are well-powered by distance and the relative abundance of total loops and well-powered loops in each 10Kb bin. The results are also summarized in text under the plots, and the same interactive plotly features are available for the plots in this tab. The legend of these plots is also interactive, and each of these groups can be removed from the plot by clicking on them in the legend.
